# Comparative evaluation of bioactive cements on biomimetic remineralization of dentin

**DOI:** 10.4317/jced.56500

**Published:** 2020-03-01

**Authors:** Nazanin Daneshpoor, Leila Pishevar

**Affiliations:** 1 Restorative dentistry specialist; 2 Department of operative dentistry, faculty of dentistry, Isfahan (khorasgan) branch, Islamic Azad university, Isfahan, Iran

## Abstract

**Background:**

This study was designed to quantitatively compare the presence of apatite peaks on demineralized dentin to induced bio-mimetically by bioactive commercial materials.

**Material and Methods:**

Dentin slice specimens (n=6) were prepared and demineralized by by17% EDTA for 2 hours. Each disks materials (Theracal, Biodentine, CPP-ACP) were freshly prepared and was maintained in close contact with a demineralized dentin specimen immersed in PBS solution during one week. To evaluation of bioactivity, cements disks (n=6) were prepared from each material and immersed in PBS solution for one week. The bioactivity and remineralization ability was evaluated using FTIR spectroscopy and Scanning Electron Microscopy. The Ca/P ratio of the surface of dentin and cements disks were compared with one -way ANOVA, independent T test and Duncan test (α=0.05).

**Results:**

Ca/P weight ratio of Biodentine (187.5) was significantly higher than Theracal (10.10) and Theracal higher than CPP-ACP (0.37) (*P*=0.008). Demineralized dentin in contact with Test materials, indicated Ca and P peak after 7 days, but not showed statistically differences between the groups (*P*=0.08).

**Conclusions:**

The outcome revealed that bioactive cements and CPP-ACP had bioactivity capability during one week. Biodentine had higher bioactivity between others. Demineralized dentin could be remineralized with bioactive materials.

** Key words:**Bioactive, cements, fourier transform infrared spectroscopy, scanning electron microscopy, tooth remineralization.

## Introduction

Either organic or inorganic constituent of dentin alter from bacterial by-products (organic acids) in the biofilm. These acids lead to dentin demineralization subsequently loss of mineral crystals, and also alter organic matrix, i.e. the collagen fibers, therefore cause dental caries (1). Contemporary treatment of carious lesions is to remove only the infected external dentin, although the affected internal dentin that can be remineralized is preserved (2).

Today, there is a challenge to remineralize remaining demineralized dentin in deep caries. Dentin remineralization is more difficult than enamel remineralization that is due to excess presence of dentin organic matrix. Most conventional carious dentin remineralzation agent involves the use of calcium and phosphate ions solution with different concentration of fluoride. Well known that common remineralization process occurs by epitaxial growth of residual apatite crystals in partially demineralized dentin. It means that there is no remineralzation, if there are a little residual crystals or none (3). Biomimetic treatment of affected caries dentin, involve guided remineralization followed by or mimicry the physiologic mechanism of tissue mineralization. This strategy is independent of growth of remaining apatite crystals so partially and well-demineralized dentin can be successfully remineralized. Bioactive glass, poly electrolytes, amorphous calcium phosphate, and calcium silicate cements are bioactive materials that has been investigated to bio-mimetically remineralize the dentin (4).

Amorphous calcium phosphate is a precursor of hydroxyapatite that lead to precipitate calcium phosphate ions, as a final sTable by-product (5). The casein phospho-peptides are resulted from tryptic digest of casein (6) and have the ability to stabilized calcium and phosphate ions through releasing small peptide sequences (CPP) resulted from partial enzymatic breakdown that led to remineralization (7,8). Casein phospho-peptide have a high binding affinity for apatite (9). Calcium silicate cements are hydrophilic materials that tolerate humidity and set in biologic solutions (blood, plasma, saliva or dentinal fluid). Wetting of tri-calcium silicate cements, cause to produce calcium silicate hydrate gel (C-S-H gel) and calcium hydroxide. Elongated setting time and the need to cover with other materials to let them fix are major deficit of the use of MTA like materials, as a pulp cap material (10). Some tri-calcium silicate base material has introduced with shorter setting time as Biodentine. Theracal, is a light cure resin modified calcium silicate cement as base and liner that designated for direct and indirect pulp cap material beneath the resin composite restorations with the goal of bond between different material layers and lower micro-leakage. It has shown that release higher amount of calcium comparison to MTA and Dycal, therefore it has the ability to alkalizing the surrounding environment (11).

The purpose of this study is to gain insight in calcium silicate cements (Biodentine and Theracal) and CPP-ACP with each other in terms of bioactivity and remineralizing ability of demineralized dentin within the short period (7 days’ immersion time) in phosphate-contained solution (human like situation). Bioactivity and remineralization ability of this three-mentioned materials have been separately analyzed before, but the novelty of this study is the quantitative comparison of these properties together. The null hypothesis is that there are no differences between bioactivity of test materials and there are no differences between them in terms of induction of remineralization within demineralized dentin.

## Material and Methods

This study gathered the number 23810201952008 as approved by the medical ethics committee of Isfahan Azad University.

-Materials: Three commercial materials (CPP-ACP, Biodentine, Theracal) were tested in this study. For the preparation of each disk material, Teflon molds (5 mm diameter and 2 mm thickness) were used. Theracal (Bisco, Schaumburg, IL, USA, LOT 1700002279) was injected to the mold, slightly pressed with a sliding glass and light cured with LED system (1200 mW/cm2, Demetron Demi, Kerr, USA) for 20 secs from each side. Each Theracal disk had 0.13 gr weight. Biodentine (Septodont, Saint Maur-des-Fossec, France, LOT B19093) was prepared following manufacturer’s instructions (five drops of liquid added to powder and triturate in amalgamator for 30 seconds) then condensed the cement in mold with a plastic spatula (provided with Biodentine, Septodont) and left to set until 12 min. each Biodentine disk had 0.15 gr weight. The construction of CPP-ACP disk, 5% weight ratio of CPP-ACP (tooth mousse, GC corporation, Tokyo, Japan, LOT 160804S) added to the formulation conventional glass ionomer luting cement (Fuji VII, GC corporation, Tokyo, Japan, LOT 160527A) in order to set this material as a disk like specimen and evaluating the bioactivity of material (12). This prepared paste was placed in the mold and let to set. Each disk had a 0.11 gr weight.

-Bio- property analysis:

Apatite deposition on the surface of material disks (Apatite forming ability test): the apatite forming ability (bio- inter activity/ bioactivity) was studied by evaluation of apatite formation on disk material in the presence of a simulated body fluid (SBF) that in this study the PBS (Phosphate Buffer Saline) was used. PBS is a physiologic saline solution with 7.4 pH, Ca and Mg free, containing some salts (4.18 K+, 152.9 Na+, 139.5 Cl- and 9.56 Po4-3 (1.5 H2PO4- and 8.06 HPO42-)). Buffering acid-base was added to preserve the pH. Salt concentration of this solution was considered as body fluids (isotonic) like plasma (13).

After preparation of each disk material (n=6 in each group), soaked them in PBS solution separately and kept in plastic container (3cm height and 4 cm diameter) at 37°C until 7 days (a PBS/cement ratio of 17 ml/gr was used). Then surface chemistry (surface component and elemental distribution of phases) was studied in dry condition by SEM-EDX and ATR-FTIR Spectroscopy methods.

Dentin Remineralization Test (DRT): Remineralization property (Bio-remineralizaton) was studied by the protocol (explains as following) of ability to induce the formation of apatite on previously demineralized dentin (13).

Dentinal samples were collected from healthy human third Molar. The teeth were kept in 0.5% thymol in 4°c for a maximum 3 months’ duration. Dentinal slices with 1± 0.1 mm thickness were prepared by an automatic cutting-instrument (CNC cutting machine, Tabriz, Iran). These sections were parallel to CEJ of tooth and done by a new diamond-cutting disk with cooling agent. All samples were checked in terms of any defects like crack or hole or presence of enamel or pulp tissue by means of optical microscopy. Therefore, the samples were completely covered by smear layer and kept in deionized water, in refrigerator before use. The surface of dentinal slices was evaluating by EDX and FTIR. Then putted the samples in 15 ml 17% EDTA (Master dent, Dentonics Inc., USA, LOT 9515) for 2 h (at room temperature) to demineralized the samples (14). Then washed them with deionized water several time and randomly categorized to four groups (n=6 in each group). Disks of set material (CPP-ACP, Biodentine, Theracal) were prepared by Teflon mold (5ml diameter and 2mm thickness). Each disk of set material was maintained in close contact with demineralized dentin slices using an orthodontic elastic band and soaked in 15ml PBS solution in plastic container and in 37°C for 7 days. This technique allowed the dentinal slices readily away from disk material after spent soaking time. One group of demineralized dentin was maintained in PBS without any treatment as a control group. After separation, slices were rinsed with deionized water, dried with ethanol, and analyzed.

-Measurement

SEM-EDX: samples were analyzed with environmental Scanning Electron Microscopy connected to a secondary electron detection for Energy Dispersive Xray analysis EDX [FIE ESEM, Quanta,200, USA] using an accelerating voltage 25 Kv. Elemental analysis (weight% and atomic %) of samples was done by using ZAF correction method. EDX was performed on dry material disk surface and dentinal slices. Dental samples need to be coated with gold for subtle discovery. Both surface of dentinal slices was analyzed (surface in contact with material [or upper] and opposite [or free] surface).

ATR-FTIR: Infrared spectra were recorded by using FTIR Spectroscopy (BURKER FT-IR, TENSOR27) equipped with attenuated total reflectance (ATR) accessory and DTGS detector for analysis of phase composition and crystal structure. The spectral resolution was 4 cm-1 and 64 the number of scan for each spectrum in the region of 400-4000 cm-1. The diameter of ATR accessory was 2 mm diameter and the IR penetration power was about 2 µm. due to high absorption of IR spectra in water, samples were dried with desiccator before spectroscopy. To minimize the problems due to inhomogeneity, five IR spectra were done at five point of different surface of each specimen.

-Statistical analysis

Bioactivity and remineralizing ability data were statistically analyzed by one -way ANOVA Test, independent T –Test and Duncan Test with 22 version of SPSS software package.

## Results

-Apatite deposition on the surface of material disks:

SEM-EDX analysis: freshly prepared material SEM analysis showed the presence of constituent elements. After 7 days’ material soaked in PBS, SEM revealed the appearance of fine spheres on the surface of Biodentine and Theracal disks and in the lesser amount on CPP-ACP disks. In addition, Ca and *P* peak of the upper surface was a sign of the presence of Ca-P deposits. The decrease in Si peak could suggest a major gathering of this element in the subsurface of CSH (Calcium silicate hydrate) phase and releasing to the solution. T independent test showed that: 1) the mean *P* peak in three materials were significantly higher after 7 –days soaking time than fresh materials. 2) The mean Ca peak in three materials was not significantly altered after 7 –days soaking time. 3) The average of Ca/P weight ratio in Biodentine group was significantly higher than fresh material after 7 days soaking time (*P*<0.05) but in CPP-ACP and Theracal groups after 7 days was less than fresh ones (*P*<0.05) (Table 1). The average of *P* peak in fresh and 7days CPP-ACP groups were significantly higher than other groups (*P*<0.05).

**Table 1:**
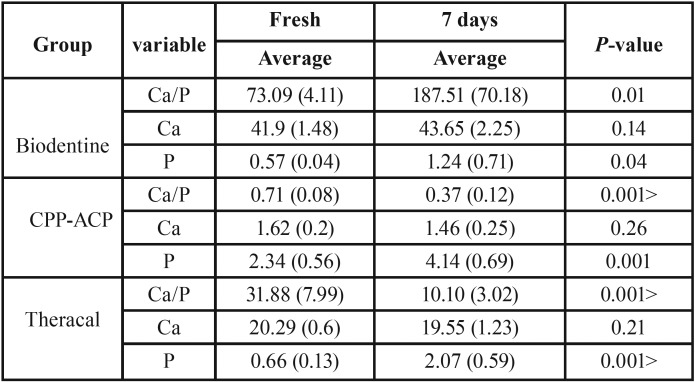
The average of Ca/P weight ratio, Calcium and phosphor wt % on test material disks separated by time analyzed by EDX.

**Figure 1:**
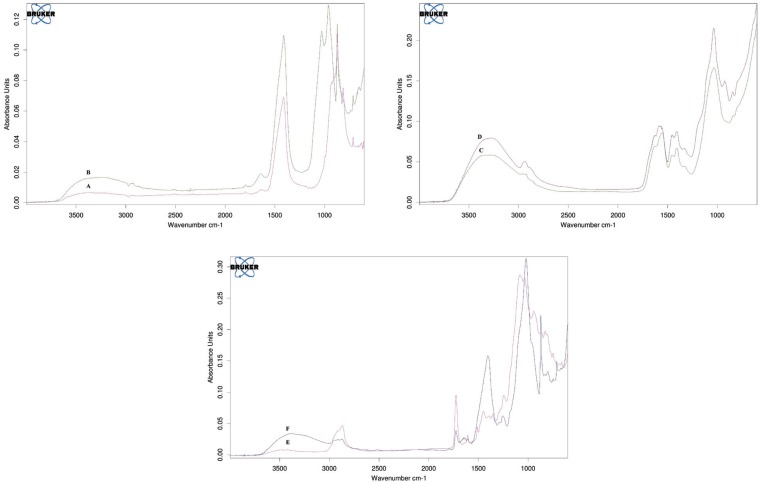
FTIR analysis of fresh (A) and 7 days (B) Biodentine disks, fresh (C) and 7 days (D) CPP-ACP disks and fresh (E) and 7 days (F) Theracal disks.

FTIR Spectroscopy analysis: Figure 1 shows the IR spectra recorded from the surface of fresh material and 7 days’ disks in PBS. Freshly prepared Biodentine and Theracal disks revealed bands related to belite, C-S-H and commencement of portlandite and anhydrite or gypsum (calcium sulfate). The elite band is not seen. In fresh CPP-ACP, bands due to the reaction of carboxylic acid with Al, Ca and formation of carboxylate salts was seen and P band due to phosphate of casein phospho-peptide amorphous calcium phosphate added to Glass ionomer was seen. After 7 days on surface of Biodentine and Theracal disks, band due to carbonate ion characteristics in the form of different chemical phase (calcite, aragonite) and type B apatite carbonate (indeed the common Ca-P band) appeared. No band due to primary cement component was seen that suggested apatite formation layer that has enough thickness (2µm) to cover the surface of cement (average penetration depth of IR spectra with the use of diamond crystal in ATR technique is 2 µm). At the end of immersion period in CPP-ACP group: 1- P-peak relative intensity increase., 2- band due to carboxylate salt was seen meaning thin apatite layer formed on cement surface so cement component is still recognizable., 3- commencement of small C=O peak showed the bioactivity and little amount of apatite formation.

FTIR analysis proved: 1- presence of carbonate apatite on the surface of cement after 7 days of being soaked in PBS. 2- More prominent apatite phase band (a relative increase of phosphate and carbonate -band peak intensity) after 7 days in Biodentine. 3- Relative increase of P peak on the surface of CPP-ACP after 7 days.

-Dentin remineralization test.

SEM-EDX analysis: There are some Ca, P, C and O peak in the intact dentin surface. Results confirmed that the demineralization treatment (17% EDTA, 2h) is effective and can remove the mineralized phase. Indeed, P & Ca peak was removed and only water and collagen/protein matrix were left on the surface.

Demineralized dentin, without any treatment, was soaked in the PBS solution for 7 days to ensure that no remineralization occurred during soaking time. Data revealed that no significant increase in Ca-P peak or remineralization occurred. One-way ANOVA showed that the average amount of Ca, P & Ca/P ratio was different (*P*<0.05) between 3 groups (intact dentin, demineralized dentin, control group). Duncan test showed that Ca, P & Ca/P ratio in the intact group was significantly higher than two other groups, but between the two, there are no differences (Table 2).

**Table 2:**
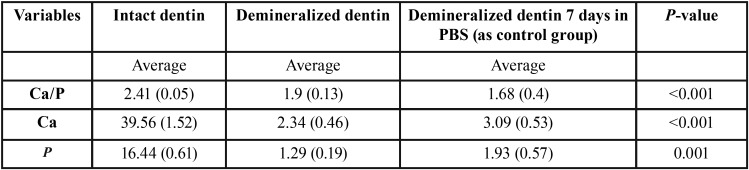
Average of calcium, phosphor and Ca/P wt % on the surface of dentin slices.

Demineralized dentin in contact with Biodentine, CPP-ACP, Theracal after 7 days of being soaked in PBS: according to EDX, Ca and P peak found on the surface, it means that remineralization occurred. Another finding in SEM images was the presence of apatite deposition in forms of fine intra tubular spheres on the treated side of dentin.

According to Table 3, independent t-test showed that the average amount of Ca, P and Ca/P ratio in 3 groups was significantly higher than the opposite surface (un-treated surface). According to Table 4, one-way ANOVA showed that the average amount of Ca, P & Ca/P ratio in the opposite surface between 3 groups have no significant differences (*P*>0.05). Ca/P ratio on the Biodentine, Theracal, and CPP-ACP-treated side was 2.87, 2.18, and 2.5 respectively that there are no statically significant differences between them (*P*>0.05).

**Table 3:**
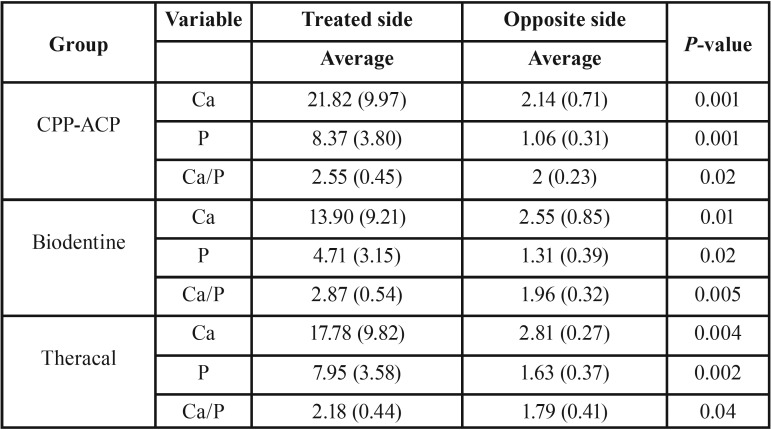
Average amount of Ca, P and Ca/P wt ratio in treated and opposite side separated bygroups.

**Table 4:**
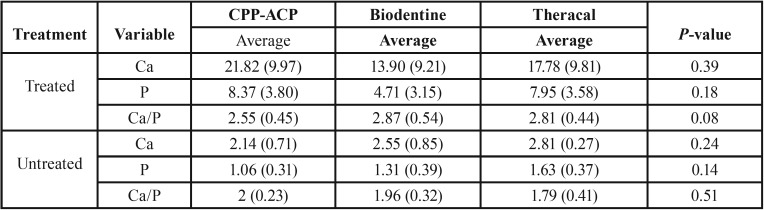
Average amount of Ca, P and Ca/P weight ratio in three groups by treatment situation.

FTIR analysis: finding obtained from FTIR spectroscopy revealed that EDTA was able to remove the mineral phase from the region of 1180-885 cm-1. In fact, the IR spectra recorded after demineralization only showed collagen (amide band) in 1700-1100 cm-1 region. However, spectral characteristic due to apatite was not showed.

Demineralized dentin after contact with experimental material for 7 days in PBS solution: dentin specimen with different spectral was remineralized. In the IR spectrum related to Biodentine and Theracal, carbonate and phosphate apatite formed and signed in the presence of bands in about 781, 1020, 1409 cm-1. In CPP-ACP, phosphate band related to the phase appeared in 1450 and band due to apatite revealed in 611 and 1028 cm-1.

## Discussion

A bioactive material provokes a positive response from the host. This material should be able to extract a biologic response in the interface and induce the formation of a band between material and tissue. Bioactivity concept (apatite-forming ability) has a close relation with bio interactivity i.e. ability to exchange data with the biologic system. This means that bioactive materials chemically react with body fluids in terms of tissue repair. So, the material ability to induce apatite formation on demineralized dentin (remineralization ability) is strongly related to its bio interactivity and bioactivity (15). These materials are able to form apatite on their surface in a short induction time (13). Different methods have been used to analyzing the efficacy of the remineralization process and bioactivity in dentinal tissue. Evaluating the methods can provide quantitative and qualitative data. Recent studies have evaluated the entrance of minerals to dentin through indirect qualitative analysis like Polarized Light Microscopy (16). Semi- quantitative analysis like Transverse Microradiography (17) and Transmission Electron Microscopy (TEM) (18) and Spectroscopic analysis like Raman and FTIR (19,20). However, each method has some limitations. IR and Raman are non- destructive novel techniques in the assessment of the vibrational mode of silicate, carbonate, hydroxyl, phosphate. In addition, they determine the remineralization effectiveness and the extent of the remineralization process simultaneously. IR Spectroscopy is more sensitive in the determination of CSH and portlandite phase in the presence of bismuth oxide than Raman Spectroscopy. On the other hand, elite and belite band in FTIR is more defined. EDX is a subtle and repatriation analysis method in studying calcium silicate cement and bioactive calcium phosphate materials that has an ability to detect constituent elements and components. The use of multiple analysis could get the obtained data closer to reality.

This study clearly shows that the Calcium silicate cement and CPP-ACP has bioactivity property, due to results from SEM- EDX (finding of fine spherules on the surface of materials with high Ca/P weight ratio. Moreover, according to result from FTIR, the presence of carbonate and phosphate apatite band with different peak intensity on the surface of Biodentine, Theracal and CPP-ACP confirm the apatite formation and bioactivity. The intensity of these peaks relatively in Biodentine was higher than Theracal and Theracal was higher than CPP-ACP. Therefore, the null hypothesis (there are no differences between bioactivity of materials) was rejected. Differences in composition could affect the amount and rate of apatite deposition. The major constituent of Biodentine (80.1%) is tri-calcium silicate (21). There is also calcium chloride as an accelerator. On the other hand, Theracal is consist of 45% Portland cement type III, as a source of Ca ions (11). Both materials have hydration chemical setting reaction, Biodentine hydration process is more elongated, but there is an uncompleted hydration reaction in Theracal because of light curing and fast polymerization (21). CPP-ACP added to GI, polymerized into the composition, may cause a fewer amount of Ca ions release.

All three materials after 7 days’ immersion time, show an increase in P peak, however, this peak in CPP-ACP was more both in freshly prepared and mainly after 7 days of storage (Table 1). This could be due to the constituent component, calcium phosphate and phosphate absorption from PBS solution. In CPP-ACP and Theracal groups, the average Ca/P ratio in 7 days was lower than fresh material (<0.05). This could be due to the formation of apatite and phosphate absorption from PBS solution than Biodentine. The storage period did not have a significant effect on remineralization efficacy, this early remineralization was done in one week and then progressed slowly (22). Apatite formation could be due to Ca release from Ca silicate cement under a wet condition and Ca-P been released from CPP-ACP. During soaking in fluids, Biodentine (like other calcium silicate cement) immediately release Ca ions and make an alkaline pH on the outer surface that leads to nucleation and crystallization of hydroxyapatite on cement surface (21). Ca ions react with phosphate the group in PBS solution and deposit in forms of Ca-P. Higher bioactivity of Biodentine could be due to higher alite/belite ratio. Indeed, alite release more calcium hydroxide than belite.

This study shows that tested materials in contact with demineralized surface had induced a significant remineralization after 7 days, all three materials on demineralized surface revealed Ca-P peak that is confirmed by an EDX analysis. However, between three groups there are no differences. So, the second hypothesis is accepted.

On demineralized dentin surface in contact with Biodentine and Theracal, carbonate and phosphate IR band appeared that confirmed the dentin remineralization. The appearance of IR band related to phosphate band in about 1000 cm-1 region, showed new apatite formation that approximately matched with normal dentin spectrum.

In Gandolfi study, the SEM of the fresh Biodentine surface showed a homogenous surface include fine granules with 5-micron width of Ca, Si (for tri-calcium silicate) and Cl (for calcium chloride). C, Zr and N represent zirconium oxide, carbonate and inorganic ingredients of Biodentine. After 7 days’ storage in HBSS, Zr and N peak disappeared, Si peak intensity was reduced and Na, Mg and P peak found in solution. Material surface covered with globular deposits and P peak was raised. Ca/P atomic ratio after 7 days reached 10.82 (23). In the present study, Ca, Si, Cl was seen in the primary component, but Zr, C and N were not seen. After 7 days, the Si peak diminished and P peak was increased too but Na and Mg peak was not seen. The Ca/P atomic ratio of the present study after 7 days had reached 6.85 in Biodentine and 6.89 in Theracal. These differences could be due to HBSS storage solution that has Ca in its composition.

In a Zhao *et al.* study, remineralizing effect of CPP-ACP add to Glass ionomer was analyzed. It showed that the remineralizing ability of CPP-ACP modified glass ionomer was improved (24). The present study also indicated the same result. Dissolution of ACP in solution lead to a super saturated solution, providing a nucleation site for Ca, phosphate deposition and apatite formation. With the increase of pH, more formation of COO- group (polyacrylate), form higher calcium polyacrylate.

**Figure 2:**
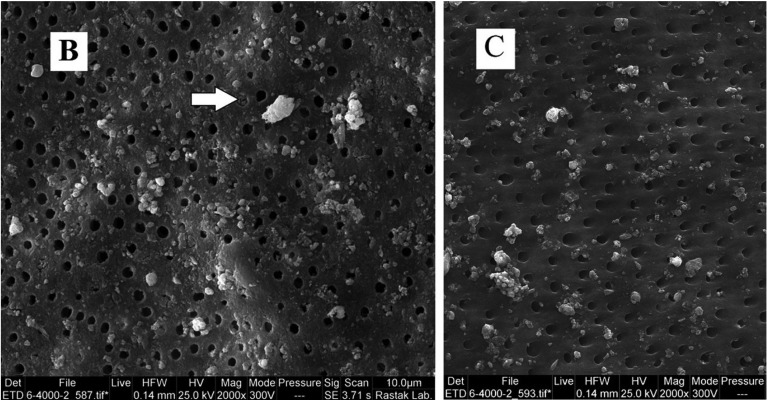
SEM image from Biodentine treated dentin. Pointer shows apatite deposition on opening of dentinal tubule and occlusion of it.

Xin li *et al.* studied chemical interplay and remineraization potential of resin -free hydraulic calcium silicate cement (hCSCs) (Biodentine and ProRoot MTA) and resin base calcium silicate cement (Theracal) at dentin through Feg-EPMA and Raman Micro Spectroscopy (22). The result was the same as the present study. All types of cements have remineralization effect after one week but any different storage period was not significant. Unlike the present study, lower remineralization was attained by resin base Theracal cement even after 6 months. In the present study SEM image revealed apatite spheroid on treated dentin tubule opening (Fig. 2) that this observation was the same with Xin Li’ observation (intra-tubular remineralization contained tubular occlusion). In Xin Li study, resin -free cement had a higher amount and rate of remineralization but this remineralization after 6 months did not reach 100 % even after six months. The differences between results may be due to the differences in methodology.

**Figure 3:**
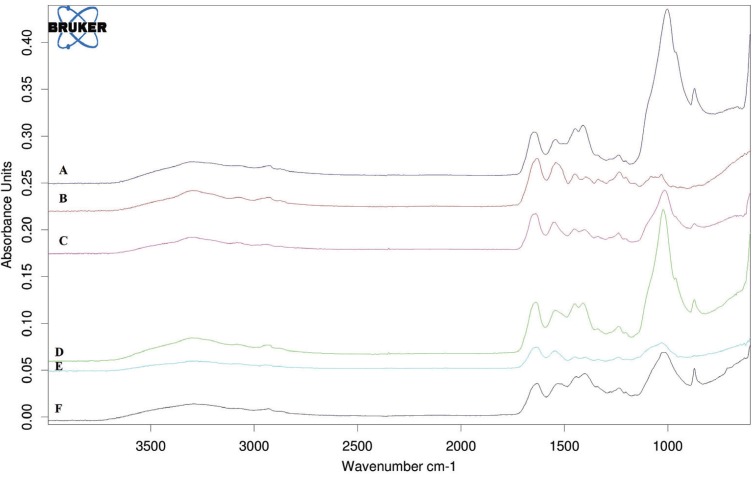
FTIR comparative analysis of intact dentin (A), demineralized dentin (B), CPP-ACP treated dentin (C), Biodentine treated dentin (D), demineralized dentin 7 days in PBS (E), TheraCal treated dentin (F).

Atmeh’s study on the remineralization potential of Biodentine and glass ionomer (Fuji IX), on completely demineralized dentin showed the mineral formation of Ca-P like Raman peak related to apatite and most florescence intensity was represented by Biodentine treated dentin (25). Like the present study, Biodentine represented matrix remineralization, intra and peritubular mineralization in BSE-SEM. In addition, the apatite peak found on normal dentin and dentin treated by Biodentine is completely similar (Fig. 3). Therefore, the formation of other Ca- P mineral components like amorphous calcium phosphate, octa calcium phosphate, di calcium phosphate di hydrate or tri calcium phosphate was implausible.

Corral Nunez *et al.* assessed the bioactive properties of Biodentine (26). They concluded that incorporation of bioactive glass Nano-particles into Biodentine improve its bioactivity, after 7 days’ immersion in SBF. The median Ca/P molar ratio of Biodentine was 15.32 (Ca=35.4 and *P* =1.8 Wt%). Biodentine formed an apatite layer on its surface and inter-tubular precipitation of deposits. At present study, the median Ca/P molar ratio of Biodentine was 15.83 (Ca=43.65 P=1.24 Wt%).

Fathy’s study on remineralization ability of Biodentine and Theracal shown that Biodentine has a higher ability to remineralize the artificial carious dentin with significantly higher mineral contents and both have significantly induced remineralization on demineralized dentin after 1 week or 6 months’ incubation period (27). According to the result of the present study, all three commercial cement had the same result in terms of remineralizing ability after 7 days (Ca=13.9, *P*=4.71 in Biodentine, Ca=17.78, P=7.95 in Theracal and Ca=21.82, P=8.37 in CPP-ACP).

Higher amounts of Ca ions in the composition and more hydration time of Biodentine (extended setting time) and enclosure of CPP-ACP by the polymeric matrix of Glass ionomer may be the reason why Biodentine has more bioactivity. Furthermore, SEM images of the surface of all three cements (with a lesser amount in CPP-ACP), revealed the formation of fine spheroid structure. SEM image of the surface of demineralized dentin that treated with Biodentine and Theracal, observed the same structure that occluded the tubule openings. The surface of CPP-ACP just revealed the deposits but certainly not on the tubules (Fig. 2), therefore, creation of a mineral zone by bioactivity of this cement may help to reminaralized the demineralized dentin.

## Conclusions

The present outcome showed that Calcium silicate cement (Biodentine and Theracal) and CPP-ACP have bioactivity property after one week. This because these materials are bio interactive and have apatite forming ability on its surface in a short period. Biodentine especially, has a higher bioactivity than others do that revealed by rapid calcium phosphate apatite deposition on SEM-EDX analysis and diagnoses of apatite band in FTIR analysis. According to SEM-EDX analysis, demineralization dentin can remineralize by bioactive cement (Biodentine and Theracal) and CPP-ACP (Bio-remineralization), but there are no significant differences between the groups.
